# Investigate the relationship between the microbiota awareness in first trimester and high risk pregnancy in Turkish women: cross-sectional study

**DOI:** 10.1186/s12884-023-06121-3

**Published:** 2023-11-21

**Authors:** Tuğba Küçükkasap Cömert, Sinan Karadeniz, Funda Akpınar

**Affiliations:** 1 Department of Nutrition and Dietetics, Gülhane Faculty of Health Sciences, University of Health Sciences Turkey, Ankara, Turkey; 2 Department of Obstetrics and Gynecology, University of Health Sciences Turkey, Ankara, Turkey

**Keywords:** Microbiota, Pregnancy, Birth weight

## Abstract

**Background:**

It is suggested that pregnancy risks may be related to microbial dysbiosis, and it is known that knowledge on this subject is reflected in behaviors. The purpose of this study was to investigate whether microbiota awareness in the first trimester of pregnancy is associated with pregnancy-related risks.

**Methods:**

Within the scope of the study, the microbiota awareness scale was administered to 426 individuals in the first trimester of pregnancy, and information on any diagnosis related to high-risk pregnancy, gestational age, birth weight, and birth height of the newborn was obtained from their file records.

**Results:**

The mean total microbiota awareness score of individuals was 61.38 ± 11.00 (26.00–91.00). The microbiota awareness score (56.85 ± 11.65) was found to be lower in individuals diagnosed with high-risk pregnancy (*p* < 0.05) than in healthy subjects (63.64 ± 9.94). Moreover, in individuals with high-risk pregnancies, a positive correlation was found between the microbiota awareness score and newborn birth weight and height (*p* < 0.05).

**Conclusion:**

The poor microbiota awareness level in pregnant women is associated with high-risk pregnancy and neonatal growth status.

## Background

In the human body, microbiota confer immunity against various diseases by acting as a physical barrier against pathogens, preventing colonization by consuming available nutrients and producing antimicrobial substances. The microbiota in the gastrointestinal tract (GIT) consists of more than 35,000 bacterial species, the majority of which are obligate anaerobes [1].

Hormonal changes, the modulation of the immune system, and metabolic adaptations that occur during pregnancy directly and indirectly affect the microbiome [2]. It has been reported that progesterone especially affects *Bifidobacterium* levels, and increased Bifidobacteria levels have been directly associated with progesterone in human and animal studies [3]. In the last period of pregnancy, an increase in beta-diversity and a decrease in alpha-diversity levels were observed, and the deterioration in the abundance of opportunistic pathogens that would affect the immune systems of newborns was also noted [4].

In addition to pregnancy-related changes, it is well documented that factors such as prepregnancy obesity, weight gain during pregnancy [5] and inflammatory bowel disease [6] can change the microbial composition, affect the neonatal microbiota and are associated with increased disease risks [7, 8]. Prepregnancy factors cause microbial dysbiosis, and microbial dysbiosis predisposes patients to pregnancy complications such as gestational diabetes, preeclampsia, infection, and premature delivery [2].

Studies have shown that individuals who have an understanding of the interactions between their microbiota and their health can make healthier lifestyle choices, such as consuming more dietary fiber [9], exercising regularly [10], or rationally using antibiotics [11]. This is also supported by studies that measured knowledge on health-related issues such as antibiotic use or preventive measures against infectious diseases and showed that knowledge on a particular issue was usually manifested in behaviors and attitudes [12, 13]. It is expected that knowledge of microbiological concepts, including the microbiota, in pregnant individuals can guide decision-making [14].

Studies on microbiota knowledge mainly focus on the therapeutic aspects of microbiota, such as probiotics [15, 16] and fecal microbiota transplantation [17, 18], and the samples mostly consist of health professionals and students. In this descriptive cross-sectional study, we aimed to measure the knowledge levels of individuals who were followed up during pregnancy to understand how they perceived the microbiota and to reveal the relationship of their attitudes toward the subject with high-risk pregnancy.

## Methods

### Sampling and participant recruitment

This study included women aged > 18 years who were in the first trimester of pregnancy and signed the informed consent form. Women aged < 18 years and in the second or third trimester of pregnancy were excluded from the study.

The sample size was determined with the calculation formula used in cases where the population was not known. Accordingly, a sample of at least 386 women was determined with the formula *n* = t² pq/d² (1.96)²× (0.50)×(0.50) ÷ (0.05)² (95% reliability).

Thus, the study was conducted with 426 individuals in the first trimester of pregnancy who applied to the Obstetrics and Gynecology Hospital in Ankara and were followed up during pregnancy.

### Study design

Anthropometric measurements (body weight, height) of the participants were taken by methods in accordance with the standards. Information regarding general characteristics (14 items) and the Microbiota Awareness Scale (20 items) was collected by the researchers using a face-to-face interview technique while the included pregnant women were waiting for their appointment or after the appointment and recorded.

All measurements were performed in accordance with the recommendations of the World Health Organization (WHO) [19, 20]. Body mass index (BMI) classification was made according to the WHO recommendations [21]. Individuals with a BMI of < 18.50 kg/m^2^ were classified as underweight, those with a BMI of 18.50–24.99 kg/m^2^ were classified as having a healthy weight, those with a BMI of 25.0–29.9 kg/m^2^ were classified as having mild obesity, those with a BMI of 30.00–34.99 kg/m^2^ were classified as having type I obesity, those with a BMI of 35.00–39.99 kg/m^2^ were classified as having type II obesity, and those with a BMI of ≥ 40 kg/m^2^ were classified as having type III obesity. Based on the Institute of Medicine criteria [22], the recommended values for gestational weight gain based on prepregnancy BMI are 12.5–18 kg for women who are underweight, 11.5–16 kg for women who have a healthy weight, 7–11.5 kg for women who are overweight, and 5–9 kg for women who are obese before pregnancy. Prepregnancy body weight was obtained from the hospital electronic database.

The “Microbiota Awareness Scale”, developed by Külcü [23], for which Turkish validity and reliability studies were conducted, was used to evaluate microbiota awareness. The scale has a minimum score of 18 and a maximum score of 100 and consists of 20 items and four subdimensions (general information, product information, chronic diseases, probiotics, and prebiotics). These scale items are rated on a 5-point scale ranging from 1 (strongly disagree) to 5 (strongly agree). The general information subdimension includes the following items (Items 1, 2, 4, 5, 6, and 13): “*The human body contains numerous microorganisms”, “The gut microbiota begins to form when the baby is in the womb”, “Antibiotic use negatively affects the gut microbiota”, “Deteriorations in the gut microbiota cause obesity”, “Diet is one of the important factors affecting the gut microbiota”*, and *“Breastfeeding positively affects the baby’s gut microbiota”*. The chronic diseases subdimension includes the following items (Items 8, 10, 11, 12, 14, 15, and 16): “*Changes in the microbiota are associated with bowel cancer”, “Deteriorations in the gut microbiota cause diabetes”, “I think the use of probiotics can solve the problem of diarrhea”, “An increase in the number of harmful bacteria in the intestines can cause nonalcoholic fatty liver disease”, “Changes in the gut microbiota are associated with celiac disease”, “I think the use of probiotics can solve the problem of constipation”*, and *“There is a relationship between gut microbiota and depression and Alzheimer’s disease”.* The probiotics and prebiotics subdimension includes the following items (Items 3, 7, and 9): “*I know what prebiotic products are*”, “*I know what probiotic products are”*, and *“Probiotics should be consumed regularly*”. The product information subdimension includes the following items (Items 17, 18, 19, and 20): “*Put the probiotics from the following foods in the box” and “Put the probiotics from the following foods in the box”.* The scale does not have a cutoff score, and a high score is considered to indicate a high level of microbiota awareness.

Individuals were followed during their pregnancy by using their file numbers, and the status of a high-risk pregnancy diagnosis (gestational diabetes, preeclampsia, preterm birth, oligohydramnios) and the gestational age, birth weight, and height of the newborn were obtained from the hospital electronic database.

### Statistical analysis

Statistical Package for the Social Sciences (SPSS) for Mac, version 22.0 (IBM Corp., Armonk, NY) was used for the statistical analysis. The normality distribution was evaluated using the Kolmogorov‒Smirnov test. Descriptive statistics are presented as numbers (n), percentages, and means ± standard deviations (SDs). Independent sample t tests, Pearson’s chi-square tests, and one-way analysis of variance (ANOVA) were used to determine whether there were any statistically significant differences between the groups. For correlations between variables, multiple linear regression analysis was used. The level of significance was determined to be *p* < 0.05.

## Results

The mean age was 28.28 ± 5.42 years (18.0–43.0 years), and the mean prepregnancy BMI was 26.69 ± 4.87 kg/m^2^. A total of 33.3% of the individuals were diagnosed with high-risk pregnancies. The mean gestational age, birth weight, and height of the newborns were determined to be 37.91 ± 1.10 weeks, 3410.93 ± 445.15 gr, and 51.08 ± 3.12 cm, respectively.

The total number of pregnancies and live births were higher, and neonatal gestational age (weeks), birth weight (g), and birth height (cm) measurements were lower in individuals with high-risk pregnancies than in healthy pregnant women (Table 1).

**Table 1:** Data on the sociodemographic and obstetric characteristics of pregnant women and their newborns

Variable	Min-Max Values (n:426)	High-risk pregnant (*n* = 142)	Healthy pregnant (*n* = 284)	*P*-value
		X̅ ±SD	X̅ ±SD	
**Age (years)**	18.00–43.00	28.92 ± 5.89	27.96 ± 5.15	0.100
**Prepregnancy BMI (kg/m**^**2**^**)**	17.00-41.80	25.64 ± 4.69	25.01 ± 4.38	0.226
**Number of pregnancies**	0.00–7.00	2.71 ± 1.54	2.28 ± 1.33	0.005*
**Live births**	0.00–6.00	1.27 ± 1.18	0.90 ± 1.01	0.001*
**Number of miscarriages/stillbirths**	0.00–4.00	0.42 ± 0.72	0.39 ± 0.72	0.598
**Gestastional age of newborn (week)**	29.00–41.00	37.73 ± 0.76	38.26 ± 1.52	0.000*
**Newborn birth weight (gr)**	1270.00-4980.00	3134.85 ± 571.60	3548.97 ± 278.28	0.000*
**Newborn birth height (cm)**	43.00–55.00	48.74 ± 3.68	52.25 ± 1.94	0.000*

*BMI *Body mass index, *p < 0.05

A total of 2.8% of the individuals were literate, 10.3% were primary school graduates, 22.1% were secondary school graduates, 38.0% were high school graduates, 26.8% were undergraduate/graduate, 5.2% were self-employed, 80.8% were housewives, 6.3% were civil servants, and 7.7% were workers. According to prepregnancy BMI (kg/m^2^) values, 3.1% were underweight, 51.2% were normal, 31.2% were overweight, 11.0% were type I obese, 2.3% were type II obese, 1.2% were type III obese, 21.6% gained body weight below the recommendations, 49.8% gained body weight in accordance with the recommendations, and 28.6% gained body weight above the recommendations during pregnancy. When their smoking status was analyzed, it was determined that 9.4% smoked, 84.7% did not smoke, 5.9% quit smoking, 41.1% had sleeping problems, and 58.9% did not have sleeping problems. It was found that 53.5% of the individuals had a normal delivery and 46.5% had a cesarean section. Data on the sociodemographic and obstetric characteristics of pregnant women based on risky pregnancy status was given in Table 2.

**Table 2:** Data on the sociodemographic and obstetric characteristics of pregnant women based on risky pregnancy status

Variable	High risk pregnant (*n* = 142)		Healthy pregnant (*n* = 284)		*P*-value
	n	%	n	%	
**Age (years)**					0.113
** 18–21**	16	11.3	24	8.5	
** 23–26**	37	26.1	100	35.2	
** 28–31**	39	27.5	85	29.9	
** ≥32**	50	35.2	75	26.4	
**Education level**					0.000*
** Literate**	5	3.5	7	2.5	
** Primary school**	17	12.0^a^	27	9.5^b^	
** Secondary school**	46	32.4^a^	48	16.9^b^	
** High school**	54	38.0	108	38.0	
** Undergraduate/graduate**	20	14.1^a^	94	33.1^b^	
**Profession**					0.060
** Self-employed**	8	5.6	14	4.9	
** Housewife**	123	86.6	221	77.8	
** Civil servant**	6	4.2	21	7.4	
** Worker**	5	3.5	28	9.9	
**Prepregnancy BMI**					0.683
** Underweight**	5	3.5	8	2.8	
** Normal**	66	46.5	152	53.5	
** Overweight**	46	32.4	87	30.6	
** Type I obese**	20	14.1	27	9.5	
** Type II obese**	3	2.1	7	2.5	
** Type III obese**	2	1.4	3	1.1	
**Body weight gain status in accordance with the recommendations during pregnancy**					0.000*
** Below**	54	38.0^a^	38	3.4^b^	
** Normal**	40	28.2^a^	172	60.6^b^	
** Above**	48	33.8	74	26.1	
**Smoking status**					
** Yes**	14	9.9	26	9.2	0.243
** No**	123	86.6	238	83.8	
** Quit**	5	3.5	20	7.0	
**Sleeping problems**					
** Yes**	51	35.9	124	43.7	0.076
** No**	91	64.1	160	56.3	
**Birth mode**					
** Normal delivery**	53	37.3^a^	175	61.6^b^	0.000*
**Cesarean section**	89	62.7^a^	109	38.4^b^	

*BMI *Body mass index, ^a,b^: different letters indicate that the difference between groups is significant

**Fig. 1:**
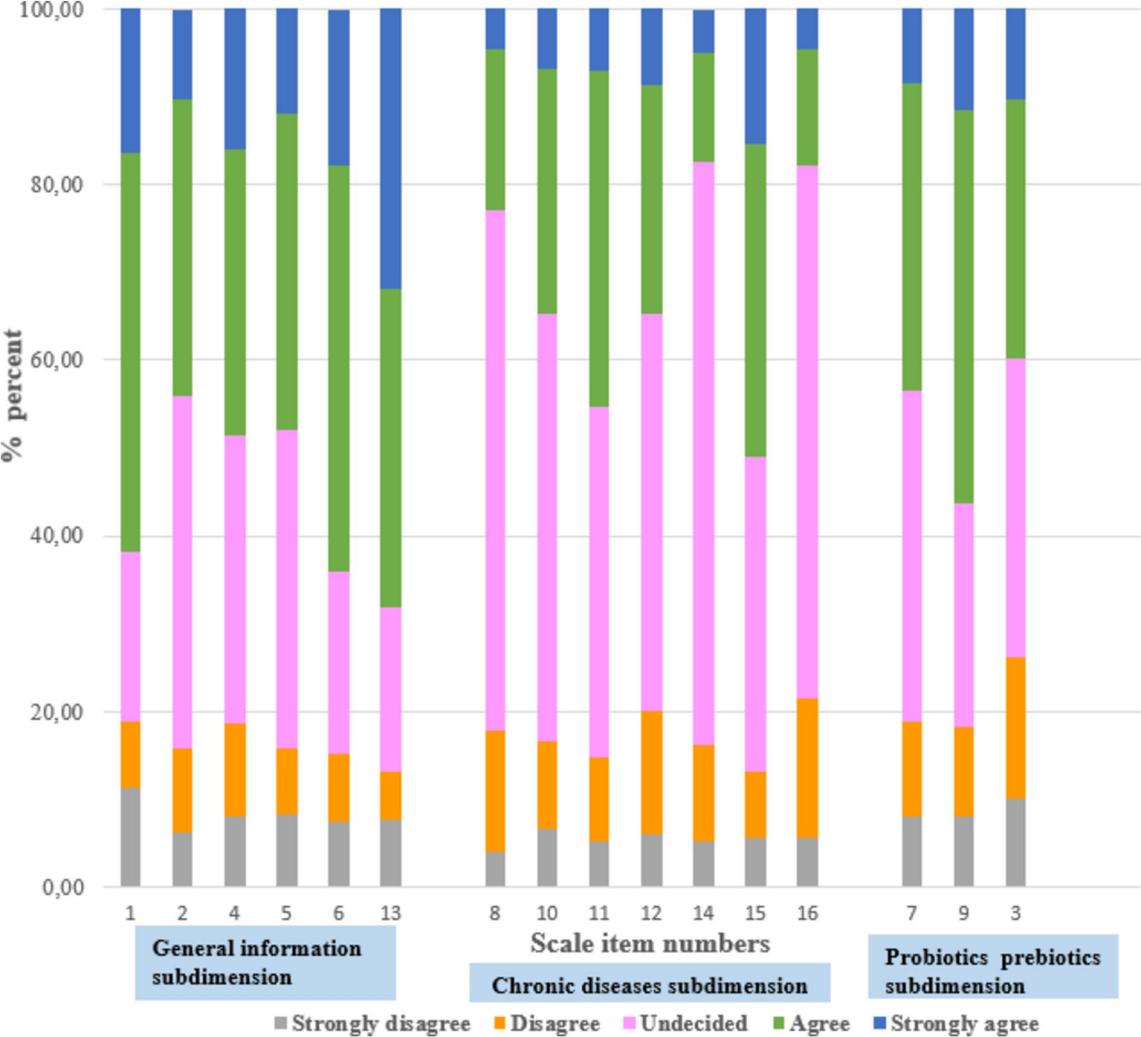
Distribution of individuals by their responses to the microbiota awareness scale (n: 426)

The distribution of individuals by their responses to the microbiota awareness scale is presented in Fig. 1. “I know what probiotic/prebiotic products are”; 87.3% of the individuals stated tea as a probiotic, and 71.1% stated banana as a prebiotic food.

The mean total microbiota awareness score of individuals was 61.38 ± 11.00 (26.00–91.00), the mean general information subscale score was 20.91 ± 4.68 (6.00–30.00), the chronic diseases subscale score was 15.37 ± 2.92 (5.00–25.00), and the probiotic and prebiotic subscale score was 16.60 ± 3.84 (5.00–25.00). A positive correlation was found between subscales and the total microbiota awareness score (*p* < 0.05).

When evaluated according to age groups, the microbiota awareness score of individuals aged between 18 and 21 years was lower than that of individuals aged between 23 and 26 and 27–31 years. Microbiota awareness scores of primary, secondary, and high school graduates were lower than those with undergraduate/postgraduate education, microbiota awareness scores of self-employed individuals were higher than those of homemakers, and those of civil servants were higher than those of homemakers. When evaluated according to the body weight gain status in accordance with the recommendations during pregnancy, it was found that individuals with insufficient body weight gain had a lower microbiota awareness score than those with appropriate body weight gain, whereas individuals with appropriate body weight gain were found to have a higher microbiota awareness score than those with excess body weight gain. Furthermore, the microbiota awareness scores of individuals who had normal delivery were found to be higher than those who had a cesarean delivery, and those who had pregnancy risks were found to have lower microbiota awareness scores than those who did not (Table 3).

**Table 3:** Microbiota awareness scores according to sociodemographic and obsetric characteristics of pregnant women (n: 426)

Variable		Microbiota awareness score (*n* = 426)	
	n	X̅ ±SD	*P*-value
**Age (years)**			0.022*
** 18–21**	40	56.32 ± 9.46^a,c^	
** 22–26**	137	61.75 ± 10.89^b^	
** 27–31**	124	61.63 ± 11.22^d^	
** ≥32**	125	62.32 ± 11.07	
**Education level**			0.000*
** Literate**	12	53.33 ± 9.38	
** Primary school**	44	55.09 ± 9.86^a^	
** Secondary school**	94	58.46 ± 8.74^c^	
** High school**	162	60.53 ± 10.58^e^	
** Undergraduate/graduate**	114	68.05 ± 10.75^b,d,f^	
**Profession**			0.000*
** Self-employed**	22	67.81 ± 10.11^a^	
** Housewife**	344	60.01 ± 10.77^b,c^	
** Civil servant**	27	70.55 ± 9.83^d^	
** Worker**	33	63.78 ± 9.73	
**Prepregnancy BMI**			0.066
** Underweight**	13	56.53 ± 11.66	
** Normal**	218	62.15 ± 10.68	
** Overweight**	133	62.10 ± 11.28	
** Type I obese**	47	57.44 ± 10.62	
** Type II obese**	10	60.40 ± 13.41	
** Type III obese**	5	60.00 ± 7.24	
**Body weight gain status in accordance with the recommendations during pregnancy**			0.000*
** Below**	92	55.83 ± 10.77^a^	
** Normal**	212	67.64 ± 8.75^b,c^	
** Above**	122	56.99 ± 9.33^d^	
**Smoking status**			0.753
** Yes**	40	60.15 ± 10.58	
** No**	361	61.48 ± 11.01	
** Quit**	25	61.80 ± 11.87	
**Sleeping problems**			0.904
** Yes**	175	61.30 ± 10.51	
** No**	251	61.43 ± 11.36	
**Birth mode**			0.000*
** Normal delivery**	228	65.43 ± 8.40^a^	
** Cesarean section**	198	56.71 ± 11.79^b^	
**Risky pregnancy status**			0.000*
** Yes**	142	56.85 ± 11.65^a^	
** No**	284	63.64 ± 9.94^b^	

In individuals with high-risk pregnancy, the microbiota awareness score was positively correlated with newborn birth weight and birth height, whereas in individuals without pregnancy risk, the microbiota awareness score was positively correlated with age and newborn birth weight (Table 4).

**Table 4:** Multiple linear regression analysis of the effect of sociodemographic and obstetric characteristics on microbiota awareness score according to the presence of pregnancy risk

Variable	Pregnancy risk status	B	T	P-value	95% confidence interval	
**Age (years)**	**Yes**	0.050	0.511	0.610	− 0.281	0.477
	**No**	0.249	3.866	0.000*	0.235	0.723
**Prepregnancy BMI (kg/m2)**	**Yes**	− 0.148	-1.700	0.091	− 0.800	0.060
	**No**	0.011	0.185	0.854	− 0.234	0.282
**Total number of pregnancies**	**Yes**	1.019	1.325	0.187	-3.793	19.177
	**No**	1.152	1.514	0.131	-2.578	19.747
**Number of live births**	**Yes**	− 0.766	-1.290	0.199	-19.099	4.018
	**No**	-1.128	-1.970	0.060	-22.105	-0.10
**Number of stillbirths/miscarriages**	**Yes**	− 0.488	-1.336	0.184	-19.366	3.754
	**No**	− 0.652	-1.552	0.122	-20.359	2.410
**Newborn gestational age (weeks)**	**Yes**	0.010	0.093	0.926	-1.566	1.721
	**No**	− 0.073	-1.259	0.209	-2.433	0.535
**Newborn birth weight (gr)**	**Yes**	0.459	3.108	0.003*	0.015	0.003
	**No**	0.118	2.037	0.043*	0.008	0.000
**Newborn birth height (cm)**	**Yes**	0.688	4.253	0.000*	1.163	3.186
	**No**	0.033	0.571	0.568	− 0.410	0.745

## Discussion

Pregnancy is a critical period in women’s lives, and many physiological, physical and spiritual changes can cause health risks [24]. Adverse conditions can endanger maternal or fetal health and result in high-risk pregnancies [25]. Mirzakhani et al. in 2020 [24] stated that almost 22% of pregnant women face high-risk pregnancies. One of the Sustainable Development Goals (SDGs) is to improve maternal health by reducing the global maternal mortality rate (MMR) to below 70 per 100,000 women by 2030 [26].

The prenatal and early postnatal periods involve dynamic changes that occur in the intestinal microbiota, and nutrition is one of the most important changeable factors affecting this process. In 2023, Cerdó et al. [27] envisaged that by creating personalized nutrition programs, possible pregnancy risks can be reduced through healthy intestinal microflora. In this regard, in 2016, Elliott et al. [28] thought that the level of knowledge about the health effects of microbiota in pregnant individuals may also affect their lifestyle choices. In this study, we aimed to evaluate the relationship between microbiota awareness in the first trimester and the diagnosis of a high-risk pregnancy.

Upon reviewing the literature, it was determined that studies evaluating microbiota/probiotic knowledge in health professionals, university students and mothers [29–32] exist, but there are no studies evaluating this knowledge in pregnant women.

In 2014, Bridgman et al. [29] analyzed the knowledge about probiotics in 413 mothers in Canada and showed that the proportion of mothers who had heard of probiotics and knew that probiotics are composed of bacteria (87.0%) was high; in 2014, Gumede et al. [30] found that more than 90% of those responsible for the nutrition of children under 5 years of age in Zimbabwe stated that fermented milk [lacto], yogurt, and fermented corn porridge were probiotics. Similarly, in Turkey in 2021, Cevik et al. [32] evaluated the level of probiotic knowledge in 519 mothers with children aged 6 months–2 years and showed that they did not have sufficient knowledge about what probiotics are, which products contain probiotics, whether there are probiotics in breast milk, and regular probiotic use. Moreover, in a study by Altındiş et al. evaluating the probiotic knowledge level of health professionals in Turkey in 2018 [33], 67.4% of individuals defined their knowledge level as moderate and 16.3% defined their knowledge level as “poor” or “very poor”. In another study evaluating the level of knowledge of probiotics, prebiotics and microbiota in obstetricians in Turkey in 2023, Tugrul Ersak et al. [34] found that only 40% of obstetricians who had been in the profession for less than 12 years thought that the use of probiotics during pregnancy was safe, while more than two-thirds who had been in the profession for 12 years or more thought that it was safe to use probiotics during pregnancy. It was found that the knowledge and attitudes about probiotics, prebiotics and microbiota may be affected by career duration.

In our study findings, the microbiota awareness scores of pregnant individuals were generally found to be moderate. It was determined that the level of knowledge about the disease-microbiota relationship (celiac disease, bowel cancer, Alzheimer’s disease) was low, while the general knowledge levels (intestinal microbiota development begins in the womb and diet is the most important factor affecting the microbiota) was better. The majority of pregnant individuals marked “undecided” in response to the statement “I know what probiotic products are” and stated that “tea is a probiotic food” (Fig. 1). In our country, probiotic teas have recently been produced and offered for sale in the market, and they have been widely promoted in the media. Natural tea does not have probiotic properties, and it is possible to interpret the reason why individuals specify tea as a probiotic as the effect of recent advertisements. Betz et al., in 2015, [35] and Alissa et al., in 2021, [36] demonstrated that the media is a key source of information about probiotics.

In another study in 2012, Cevik Guner et al. [31] found that the level of education and type of occupation of mothers in Turkey were associated with the level of probiotic knowledge. Similarly, in another study conducted by Aslan et al. in 2019 [37] the level of probiotic knowledge increased as the level of education increased in adults. Another study conducted in Canada by Bridgman et al. in 2014 [29] found that there was no difference in the educational level between mothers who gave probiotics to their children and those who did not. In our study, it was found that the microbiota awareness score was higher in pregnant women with undergraduate/graduate education levels and in those who were self-employed or civil servants than in women with primary, secondary, and high school education levels and housewives (Table 3). As the education level increased, the microbiota knowledge level increased.

In studies evaluating the level of probiotic knowledge, age has been shown to be a determining factor [29, 31, 35–37]. Barqawi et al. [38] conducted a study of individuals between the ages of 18–29 years in 2021 and Allah et al. [39] pointed out that individuals aged 18–25 years had the highest level of knowledge in 2019. In our study, the relationship between age and the microbiota awareness score in healthy pregnant women was determined. The microbiota awareness score increased with age, and it was also shown that women aged 18–21 years had a lower level of knowledge than those aged 22–26 and 27–31 years (Table 3).

In a study by Kolady et al. in 2018 [40] evaluating 1497 individuals in the United States, the probiotic awareness status of individuals with normal body weight was found to be higher than those who were underweight or overweight. In 2022, Hamurcu et al. [32] conducted a study among nutrition and dietetics department students in our country and found that the level of microbiota awareness was lower in overweight individuals. In this study, it was found that women whose weight gain complied with the recommended guidelines during pregnancy had a higher level of microbiota awareness than those whose weight gain was inadequate or excessive (Table 3). In 2019, Timmis et al. [14] suggested that providing adequate information on microbiota and explaining evidence-based data guides behavioral decisions.

The results of this study demonstrated that women who gave birth vaginally scored higher on the microbiota awareness scale than those who delivered by cesarean section (Table 3). Similarly, the microbiota awareness score of healthy pregnant women was higher than that of high-risk pregnant women. Since cesarean delivery is a preferred delivery method among women with high-risk pregnancies, both findings confirm each other. It is thought that the presence of appropriate nutritional behaviors together with the level of knowledge in individuals with high microbiota awareness helps to avoid risk factors that negatively affect the microbiota.

Maternal microbial composition changes due to metabolic and physiological factors occurring during pregnancy, and this may alter fetal growth directly or through its effects on gestational body weight gain [41]. In 2022, Cömert et al. [42] evaluated the effect of prepregnancy obesity status on maternal and meconium microbiota and fetal growth in Turkey and showed that neonatal birth weight and the birth weight/birth height ratio were associated with maternal intestinal microbiota alpha diversity indices.

Likewise, in the present study, a correlation was found between neonatal birth weight and the microbiota awareness score in both groups of women with healthy and high-risk pregnancies, and birth height was also shown to be correlated with the microbiota awareness score in women with high-risk pregnancies (Table 4).

One of the strengths of our work is its novelty. To our knowledge, this is the first study to evaluate the microbiota knowledge of pregnant women. It was also a prospective cross-sectional study, and information was collected using a face-to-face interview technique. However, the fact that the study was conducted in a single center is a limitation of the study.

## Conclusion and recommendations

It was noted in our study that high-risk pregnancy individuals had lower level of microbiota knowledge than in healthy pregnants. And the level of microbiota knowledge increases, fetal growth indicators (birth weight, height) also increase has been determined.

Knowledge is an essential component of decision-making, choice making, and behavior shaping. It is evident that initiatives to increase microbiota awareness and education on this subject will make a significant contribution. Given the high frequency of cesarean sections in our country, pregnancy risks are associated with fetal and maternal mortality, and fetal growth retardation results in high health costs. Studies have shown the profound impact of microbiota on health, and there is a need to simplify this information and transfer it to society, especially to at-risk groups. There is a need for effective education plans that include women of reproductive age starting from the prepregnancy period, increasing the level of microbiota knowledge and enabling them to turn knowledge into action.

## Data Availability

Data used and analyzed in the current study are available from the corresponding author upon reasonable request.

## Abbreviations

BMI: Body mass index
WHO: World Health Organization
SPSS: Statistical Package for the Social Sciences
SDs: Standard deviations
ANOVA: One-way analysis of variance
IOM: Institute of Medicine
SDGs: Sustainable Development Goals
MMR: Maternal mortality rate
